# Impairment of μ-calpain activation by rhTNFR:Fc reduces severe burn-induced membrane disruption in the heart

**DOI:** 10.1038/s41420-021-00810-8

**Published:** 2022-01-10

**Authors:** Meng-Shu Cao, Ting-Yan Zhao, Zhi-Long Song, Hong-Ting Lu, Yun Zheng, Xiao-Ming Gu, Tao Lu, Qiong Wang, Jing-Jun Zhou

**Affiliations:** 1 Department of Cardiology, Xijing hospital, Fourth Military Medical University, Xi’an, 710032 China; 2 Department of Physiology and Pathophysiology, Fourth Military Medical University, Xi’an, 710032 China; 3 Department of Cardiovascular Surgery, Xijing Hospital, Fourth Military Medical University, Xi’an, 710032 China

**Keywords:** Trauma, Cardiomyopathies

## Abstract

Stress cardiomyopathy is a major clinical complication after severe burn. Multiple upstream initiators have been identified; however, the downstream targets are not fully understood. This study assessed the role of the plasma membrane in this process and its relationship with the protease μ-calpain and tumor necrosis factor-alpha (TNF-α). Here, third-degree burn injury of approximately 40% of the total body surface area was established in rats. Plasma levels of LDH and cTnI and cardiac cell apoptosis increased at 0.5 h post burn, reached a peak at 6 h, and gradually declined at 24 h. This effect correlated well with not only the disruption of cytoskeletal proteins, including dystrophin and ankyrin-B, but also with the activation of μ-calpain, as indicated by the cleaved fragments of α-spectrin and membrane recruitment of the catalytic subunit CAPN1. More importantly, these alterations were diminished by blocking calpain activity with MDL28170. Burn injury markedly increased the cellular uptake of Evans blue, indicating membrane integrity disruption, and this effect was also reversed by MDL28170. Compared with those in the control group, cardiac cells in the burn plasma-treated group were more prone to damage, as indicated by a marked decrease in cell viability and increases in LDH release and apoptosis. Of note, these alterations were mitigated by CAPN1 siRNA. Moreover, after neutralizing TNF-α with rhTNFR:Fc, calpain activity was blocked, and heart function was improved. In conclusion, we identified μ-calpain as a trigger for severe burn-induced membrane disruption in the heart and provided evidence for the application of rhTNFR:Fc to inhibit calpain for cardioprotection.

## Introduction

Stress cardiomyopathy or cardiac dysfunction is a major clinical complication of severe burns [1, 2]. This condition occurs almost immediately after burn injury and reaches a nadir approximately 6−24 h post burn. Stress cardiomyopathy is characterized by impaired cardiac output, decreased mean arterial pressure, increased plasma levels of the lactate dehydrogenase (LDH) and cardiac troponin I (cTnI), and cardiac cell death in clinical and animal studies [3, 4]. Stress cardiomyopathy contributes to the development of sepsis and multiple organ failure and is associated with high morbidity and mortality in patients with extensive skin burns [4, 5].

Severe burns trigger a wide range of responses that cause stress cardiomyopathy. Some of the identified upstream initiators include (1) reduced venous return due to microvascular hyperpermeability and a 10- to 20-fold surge in plasma catecholamines, leading to hypovolemic perfusion and an increase in oxygen consumption in the heart [3]; (2) systemic proinflammatory mediator storms, including tumor necrosis factor-α, interleukin-1β, and interleukin-6, which alone or in concert, depress heart function and induce cell death [6]; and (3) bioenergetic perturbations due to insulin resistance and mitochondrial dysfunction, which predispose cardiac cells to hypoxic insult [7, 8]. Despite all this knowledge, the downstream targets remain unclear.

The plasma membrane serves as a key line of defense for cell survival [9]. The membrane-associated cytoskeleton is a central governing factor that controls the structural and functional properties of the plasma membrane, and its roles in cell fate are currently receiving attention. Dystrophin is a constituent of the dystrophin-glycoprotein complex, which links the cytoskeleton and extracellular matrix and mechanically stabilizes the plasma membrane against shear stress imposed during muscle contraction [10]. Ankyrin-B, an adaptor protein, directs membrane tethering of Na^+^ channels and the Na^+^/Ca^2+^ exchanger and plays an important role in the maintenance and control of cell excitability [11, 12]. Spectrin, also known as fodrin, functions as tetramers consisting of two α and two β subunits and confers membrane flexibility [13]. In addition, membrane-associated cytoskeletal proteins also act as hubs for the organization of various signal transduction complexes [14, 15]. To date, there is increasing evidence that mutations or cleavage of these membrane-associated cytoskeletal proteins causes membrane disruption, leading to arrhythmias and cell death in the heart [12, 16, 17]. However, the character of membrane-associated cytoskeletal proteins in severe burn-induced stress cardiomyopathy is unclear.

Calpain (CAPN) is a superfamily of Ca^2+^-activated nonlysosomal cysteine proteases consisting of at least 16 genes in eukaryotes [18]. μ-calpain, a heterodimer consisting of the large catalytic subunit CAPN1 and a small regulatory subunit, is abundant in the heart [18, 19]. We demonstrated that CAPN1 was recruited to the plasma membrane under ischemia/reperfusion stress [20]. Calpain specifically cleaves α-spectrin into 150 kDa fragments [21]. Calpain also detaches ankyrin-B and dystrophin from the plasma membrane [17]. These alterations hasten membrane integrity fragility, resulting in cell necrosis and heart dysfunction [17]. Because intracellular Ca^2+^ overload, which is required for calpain activation, occurs in cardiomyocytes after severe burn injury [3], a similar pathophysiological process may result in stress cardiomyopathy.

Another issue that is of great concern is the burst of proinflammatory cytokines after severe burns, and there has been increasing attention on the role of tumor necrosis factor alpha (TNF-α) in this context, which correlates with the severity of the burn [22, 23]. High levels of TNF-α activate NF-κB-dependent inflammatory signaling pathways, stimulate the formation of reactive oxygen species, and induce insulin resistance, all of which synergistically cause cell death or dysfunction [24–26]. Despite a comprehensive understanding of the function of TNF-α, the role of TNF-α in calpain activation in stress cardiomyopathy has not been clarified.

In this study, we first determined the involvement of membrane-associated cytoskeletal proteins and membrane integrity in severe burn-induced heart injury by altering calpain. Next, we used cultured cells to corroborate the indispensable effects of hypovolemic perfusion on cell injury and used CAPN1 siRNA to evaluate the role of μ-calpain in this process. Third, we used a recombinant human type II tumor necrosis factor receptor: IgG Fc fusion protein (rhTNFR:Fc) to neutralize TNF-α and examined calpain activity and heart function after severe burn injury.

## Results

### Burn injury caused heart damage, disrupted the membrane-associated cytoskeleton, and activated μ-calpain

**Fig. 1:**
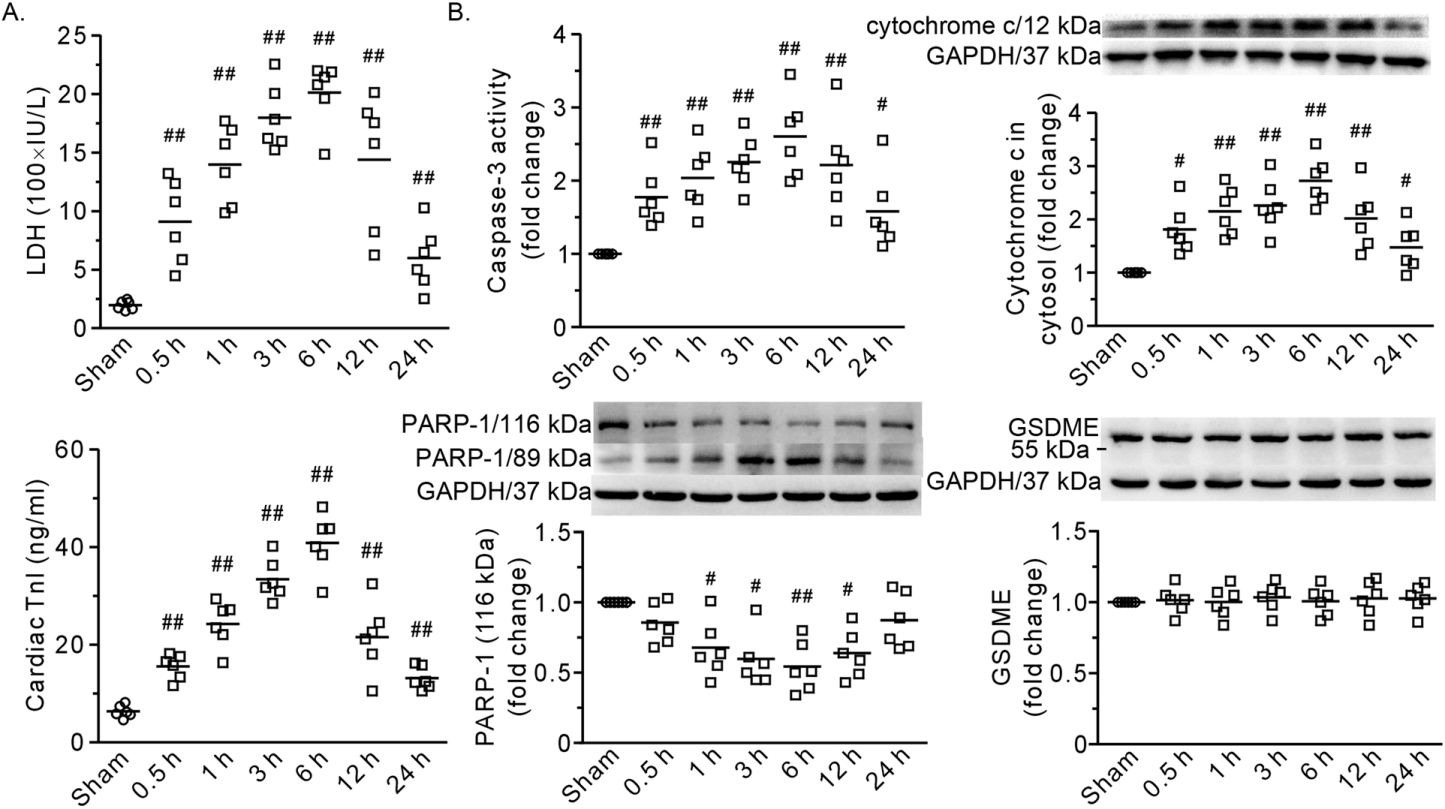
A time course of the effects of severe burns on the heart. **A** Plasma levels of LDH and cTnI. **B** The results of caspase-3 activity and representative blots and densitometric analysis of cytochrome c, PARP-1, and GSDME in the left ventricle. GAPDH served as a loading control. 0.5–24 h refers to the time point at which the heart specimens were harvested after burn injury. Each independent data with the mean is presented. *n* = 6 rats in each group. Western blotting was performed in six independent biological experiments, and there were three technical replicates per sample. ^#^*P* < 0.05, ^##^*P* < 0.01 vs. the sham group.

As shown in Fig. 1, the heart exhibited damage in the first 24 h of extensive burn injury. The plasma levels of LDH and cTnI increased at 0.5 h, reached a peak at 6 h, and gradually declined at 24 h (Fig. 1A). Both caspase-3 activity and the level of cytosolic cytochrome c were significantly increased after burn injury (Fig. 1B). Furthermore, the DNA repair enzyme PARP-1, which is 116 kDa, was apparently cleaved into fragments (89 kDa), indicating the occurrence of apoptosis (Fig. 1B), although there was no discerned cleavage of full-length GSDME, which is an indicator of pyroptosis (Fig. 1B). Taken together, these results demonstrated that extensive burn injury induced heart damage.

**Fig. 2:**
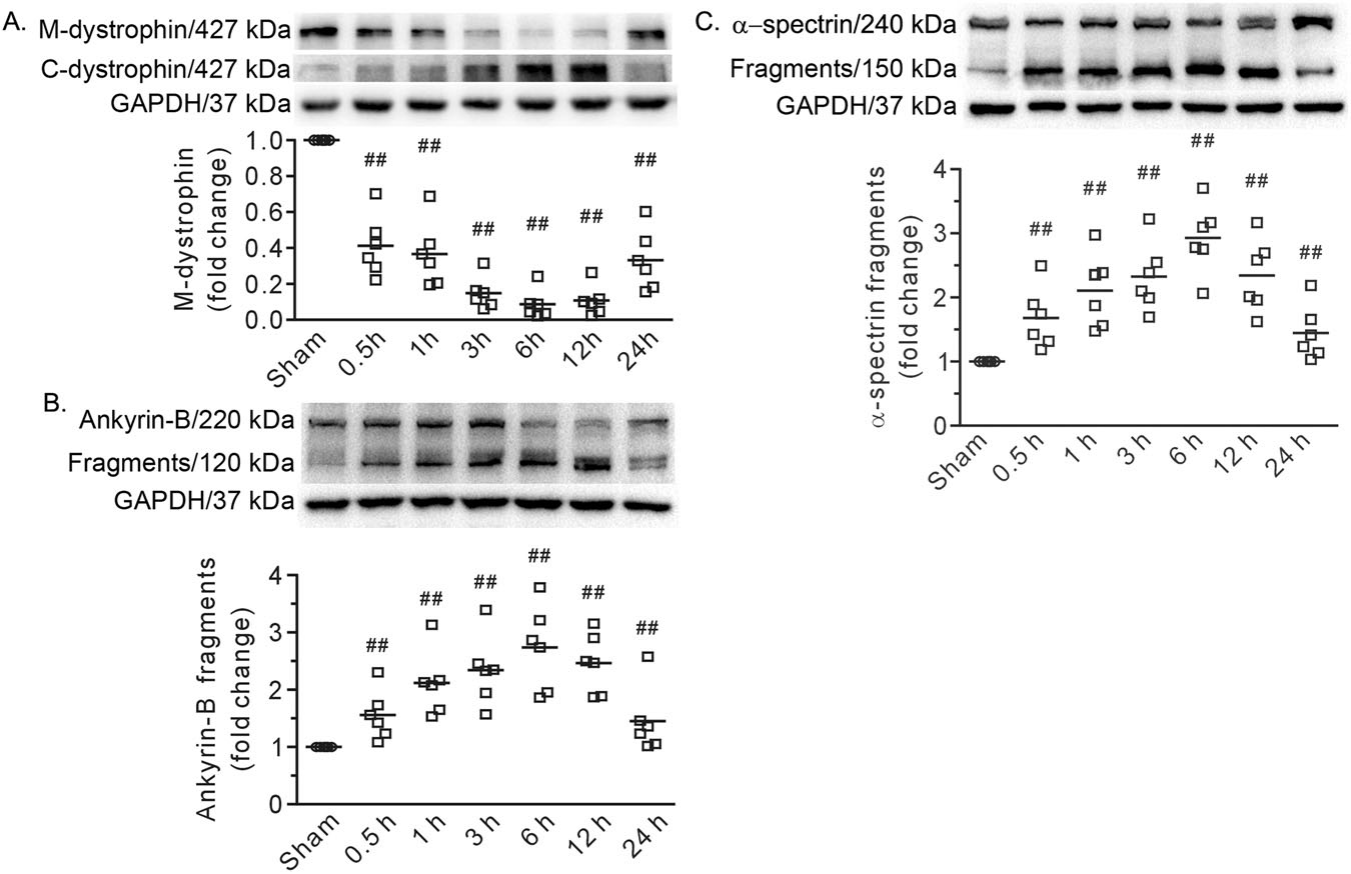
Burn injury cleaved the cytoskeleton in the heart. **A** Representative blots showing dystrophin in the membrane (M-dystrophin) fraction and cytosolic fraction (C-dystrophin), along with the densitometric analysis results. **B**, **C** Representative blots showing the cleavage of ankyrin-B and α-spectrin and the densitometric analysis results. GAPDH served as a loading control. 0.5–24 h refers to the time point at which the heart specimens were harvested after burn injury. Each independent data with the mean is presented. *n* = 6 rats in each group. Western blotting was performed in six independent biological experiments, and there were three technical replicates per sample. ^##^*P* < 0.01 vs. the sham group.

**Fig. 3:**
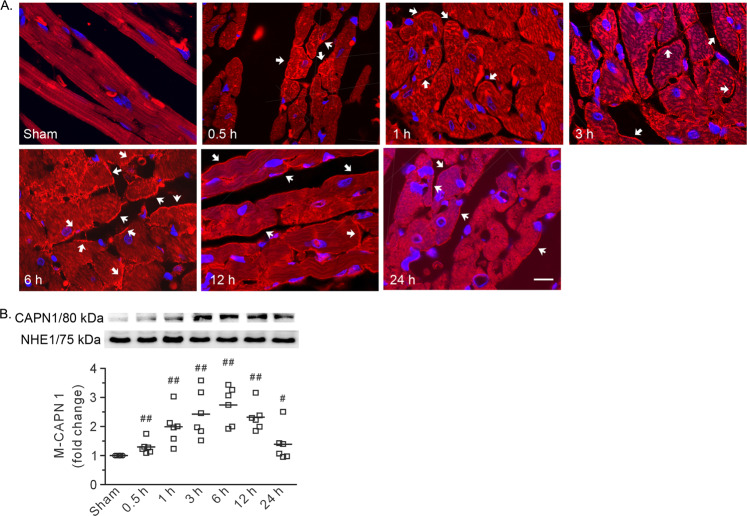
Burn injury induced plasma membrane recruitment of CAPN1 in the heart. **A** Representative confocal images. Arrows denote the positive signals of CAPN1 on the membrane. 0.5–24 h refers to the time point at which the heart specimens were harvested after burn injury. Scale bar, 10 μm. **B** Representative blots showing CAPN1 in the membrane fraction and the densitometric analysis results. Each independent data with the mean is presented. *n* = 6 rats in each group. Western blotting was performed in six independent biological experiments, and there were three technical replicates per sample. ^#^*P* < 0.05, ^##^*P* < 0.01 vs. the sham group.

Western blot analysis revealed that the level of dystrophin in the plasma membrane was dramatically decreased (Fig. 2A). Similarly, the levels of the 120 and 150 kDa fragments of ankyrin-B and α-spectrin, respectively, were strikingly increased (Fig. 2B, C). Furthermore, the time course of the changes in these proteins was quite similar to that in heart injury (Figs. 1 and 2). As the 150 kDa fragment of α-spectrin indicates calpain activation, we measured CAPN1 in the heart. Confocal images revealed plasma membrane recruitment of CAPN1 after severe burn injury, and Western blot analysis showed that the expression of CAPN1 on the membrane increased at 0.5 h, peaked at 6 h, and declined at 24 h (Fig. 3A, B). Of note, the changes were similar to those in heart injury and cytoskeleton disruption. These data indicate that disruption of the membrane-associated cytoskeleton by μ-calpain is involved in heart damage after burn injury.

### The calpain inhibitor MDL28170 reduced cell damage, improved heart function, and protected cardiac cell membrane integrity against burn stress

**Fig. 4:**
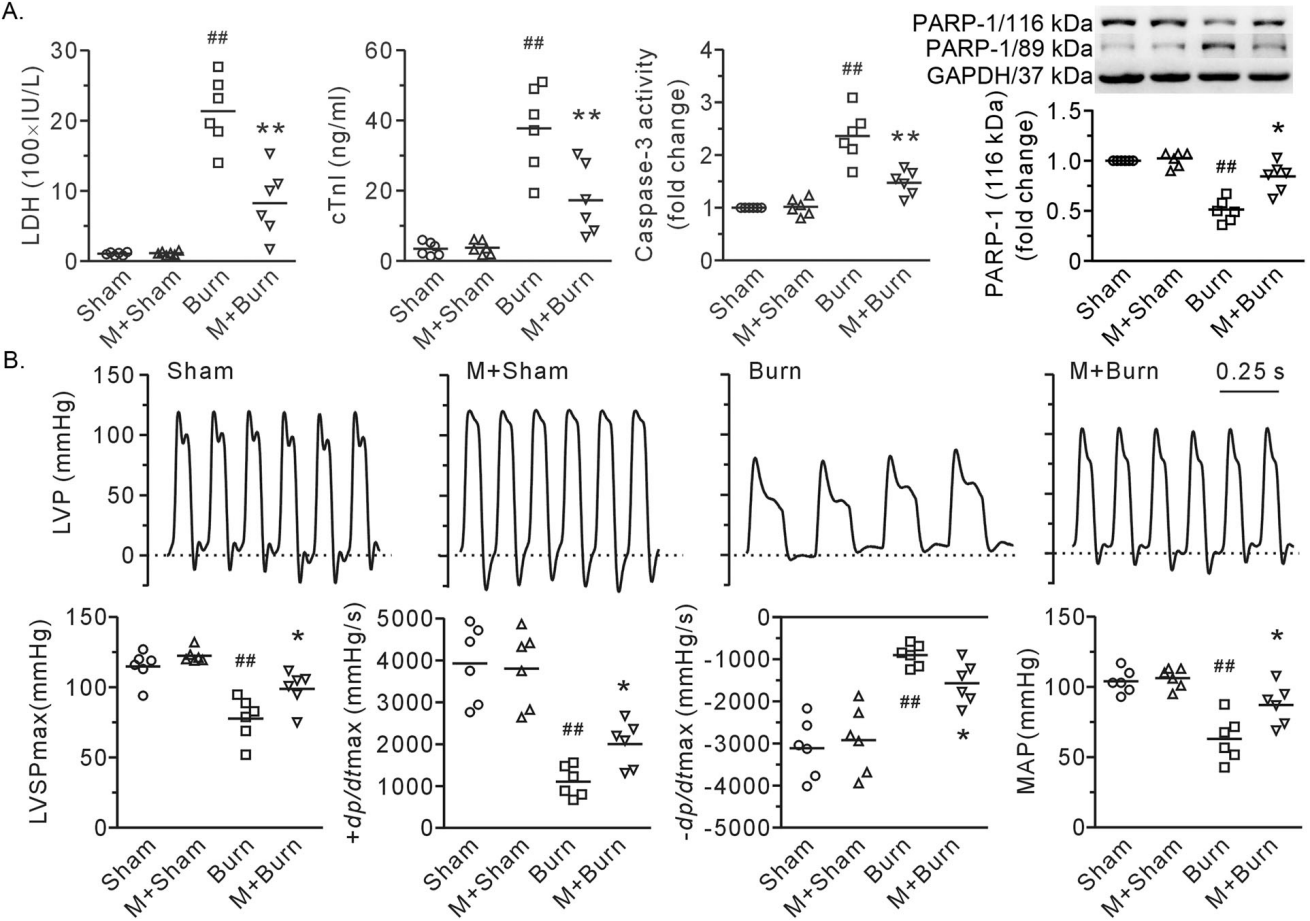
Intravenous administration of MDL28170 (3 mg/kg. b. w.) ameliorated burn injury-induced heart damage. **A** The plasma levels of LDH and cTnI, caspase-3 in heart tissue, and representative blots showing PARP-1 with densitometric analysis. GAPDH served as a loading control. **B** Representative left ventricular pressure tracings and the results of heart function and mean arterial blood pressure (MAP). Each independent data with the mean is presented. *n* = 6 rats in each group. Western blotting was performed in six independent biological experiments, and there were three technical replicates per sample. ^##^*P* < 0.01 vs. the sham group. *P < 0.05, **P < 0.01 vs. the burn group. M + Sham and M + Burn refer to the sham-operated and burn-treated rats that received MDL28170, respectively.

Compared with those in the burn group, rats treated with MDL28170 displayed salient reductions in plasma LDH and cTnI (Fig. 4A). The rats also showed reduced caspase-3 activity and PARP-1 degradation, indicating a reduction in cardiac cell apoptosis (Fig. 4A). Burn injury resulted in sharp decreases in LVSPmax, ±d*p*/d*t*max, and mean arterial pressure. Of particular interest, these alterations were diminished by injection of the calpain inhibitor MDL28170 (Fig. 4B).

**Fig. 5:**
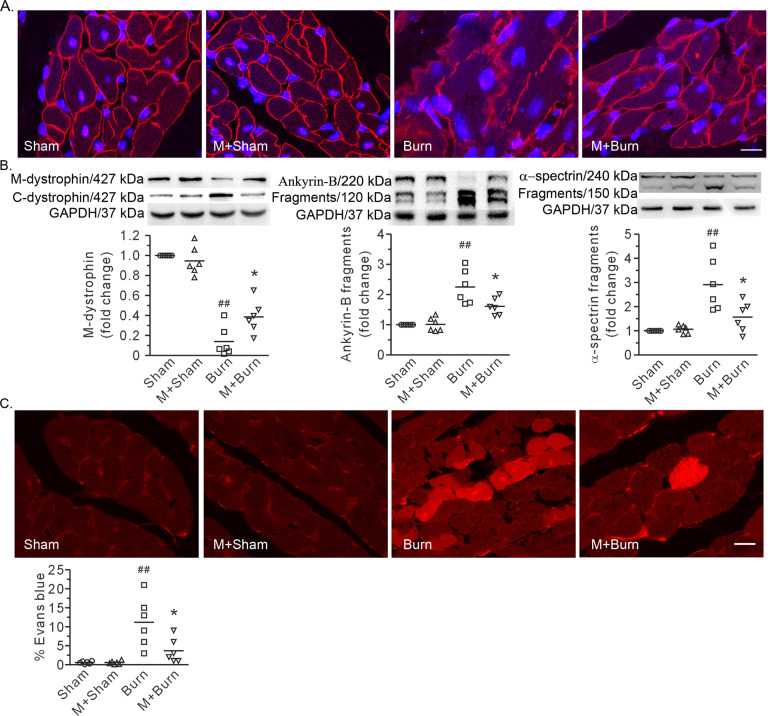
MDL28170 improved membrane-associated cytoskeleton and plasma membrane integrity in cardiomyocytes after burn. **A** Representative confocal images showing dystrophin distribution. Scale bar, 10 μm. **B** Representative blots showing dystrophin in the membrane and cytosolic fractions, full-length and fragments of ankyrin-B and α-spectrin, and the densitometric analysis results. GAPDH served as a loading control. The ratio of the membrane level to total level or the ratio of the fragment to the total signal per lane is normalized to that in the sham group. **C** Representative images showing cell uptake of Evans blue and quantification of the positive area. Scale bar, 10 μm. Each independent data with the mean is presented. *n* = 6 rats in each group. Western blotting was performed in six independent biological experiments, and there were three technical replicates per sample. ^##^*P* < 0.01 vs. the sham group. *P < 0.05 vs. the burn group.

Immunofluorescence images and Western blot analysis revealed that MDL28170 blunted the burn injury-induced loss of dystrophin in the plasma membrane in the heart (Fig. 5A, B). Furthermore, MDL28170 also alleviated the proteolysis of ankyrin-B and α-spectrin, which are two membrane-associated cytoskeletal proteins (Fig. 5B). Burn injury caused a noticeable increase in cardiac cell uptake of Evans blue, indicating the disruption of membrane integrity (Fig. 5C). More importantly, this effect was reversed by MDL28170 (Fig. 5C). Finally, MDL28170 had no discernible effects on sham-operated rats (Figs. 4 and 5).

### CAPN1 siRNA blunted burn plasma-induced cardiac cell shock

**Fig. 6:**
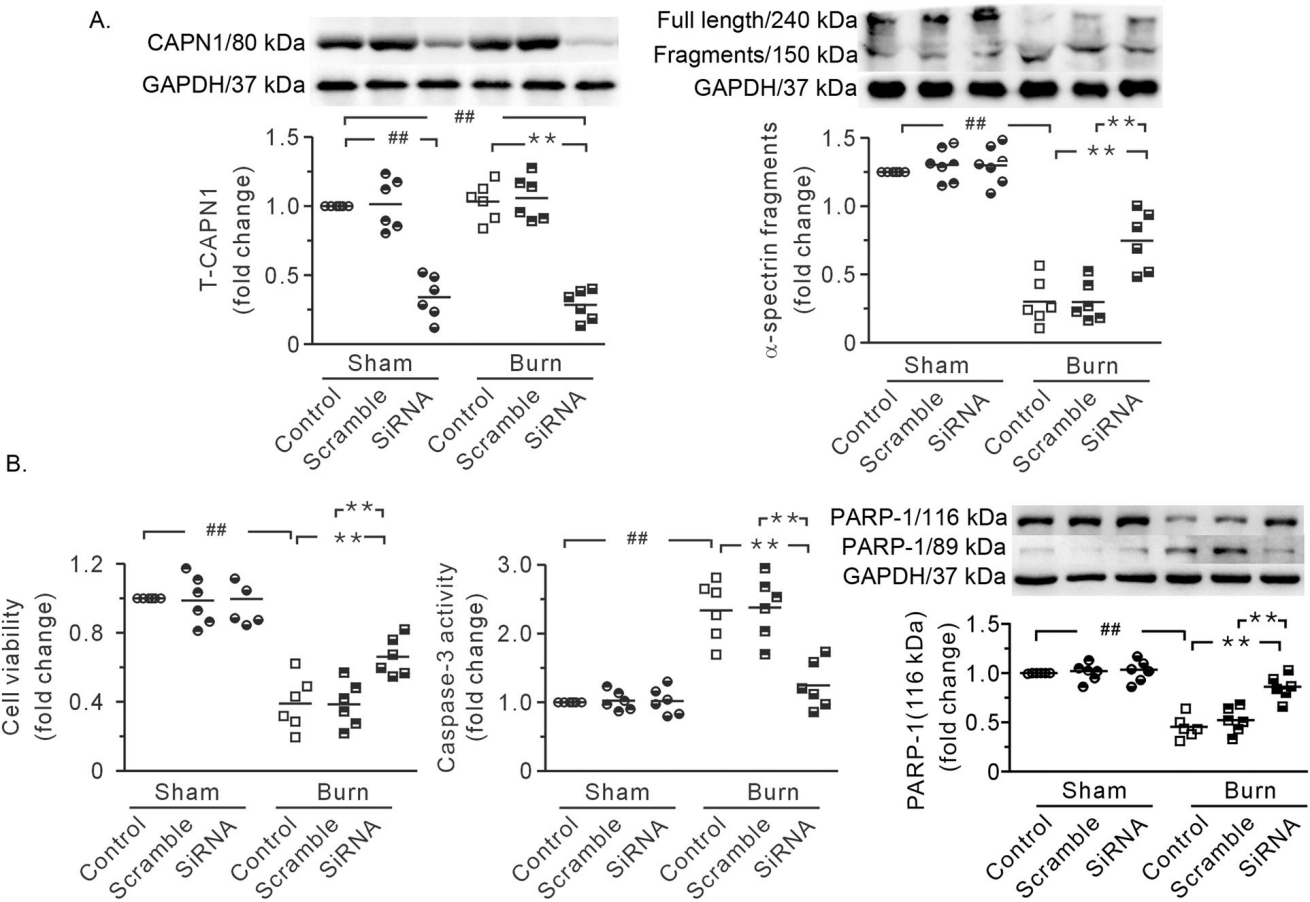
CAPN1 siRNA ameliorated burn plasma-induced cardiac cell injury. **A** Representative blots and densitometric analysis showing siRNA-mediated knockdown of CAPN1 and the proteolysis of α-spectrin. **B** Blockade of CAPN1 with siRNA improved cell viability and reduced caspase-3 activity and PARP-1 degradation. GAPDH served as a loading control. Each independent data with the mean is presented. *n* = 6. Western blotting was performed in six independent biological experiments, and there were three technical replicates per sample. ^##,^ **P < 0.01.

To rule out the possibility that cardiac cell injury was not due to hypovolemic perfusion in vivo and to assess the role of CAPN1 in this process, cultured neonatal cardiac cells were treated with CAPN1 siRNA and burn plasma in vitro. Transfection with CAPN1 siRNA markedly decreased CAPN1 protein levels, suggesting successful knockdown (Fig. 6A). Compared to those in the control group, cardiac cells in the burn plasma-treated group were more prone to damage after 4 h of anoxia/reoxygenation. Burn plasma caused an increase in the cleaved fragments of α-spectrin (Fig. 6A). Cells treated with burn plasma exhibited marked increases in cell injury, caspase-3 activity, and PARP-1 degradation (Fig. 6B). Of note, these alterations were blunted by CAPN1 siRNA (Fig. 6). These data revealed that μ-calpain-mediated severe burn-induced heart injury.

### RhTNFR:Fc inhibited calpain activity and mitigated severe burn-induced heart injury

**Fig. 7:**
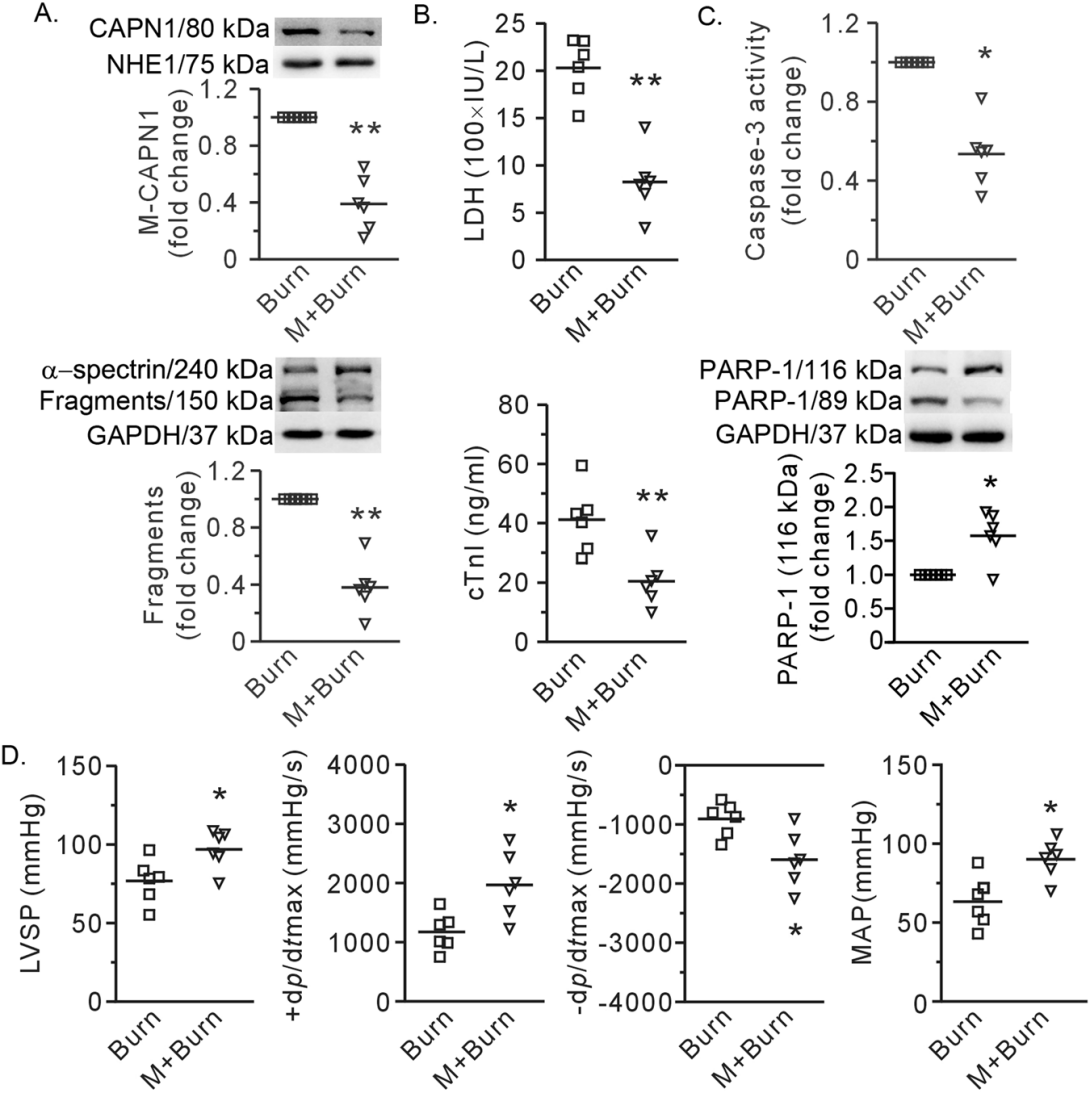
RhTNFR:Fc mitigated calpain activation and myocardial damage caused by burns. **A** Representative blots and the results showing membrane recruitment of calpain 1 (CAPN1) and the fragments of α-spectrin. NHE and GAPDH were used as the loading controls for membrane proteins and total proteins, respectively. **B** Plasma levels of LDH and cTnI. **C** The results of caspase-3 activity and representative blots and densitometric analysis of PARP-1 with GAPDH as an internal control. **D** The results showing heart function and mean arterial pressure (MAP). Each independent data with the mean is presented. *n* = 6 rats in each group. Western blotting was performed in six independent biological experiments, and there were three technical replicates per sample. *P < 0.05, **P < 0.01 vs. the burn group. M + Burn refers to rats that received rhTNFR:Fc 30 min post-burn.

The Western blot data revealed that the membrane recruitment of CAPN1 was significantly inhibited when rhTNFR:Fc (5 mg/kg b.w.) was injected intravenously 30 min after burn induction. Moreover, the cleaved fragments of α-spectrin were significantly reduced, indicating a reduction in calpain activity (Fig. 7A). More importantly, rhTNFR:Fc reduced cell death, as reflected by decreases in plasma LDH and cTnI, caspase-3 activity, and PARP-1 degradation (Fig. 7B, C). Finally, rhTNFR:Fc antagonized the decline in LVSPmax, ±d*p/*d*t*max, and MAP caused by severe burns (Fig. 7D).

## Discussion

The most important findings in this study were that (1) blocking calpain alleviated disruptions in membrane-associated cytoskeletal proteins, including spectrin, ankyrin-B and dystrophin, and membrane integrity in the heart after severe burn; (2) knockdown of CAPN1, a catalytic subunit of μ-calpain, mitigated burn stress-induced cardiac cell injury; and (3) treatment with rhTNFR:Fc inhibited calpain activity and mitigated severe burn-induced heart injury. These data, for the first time, reveal μ-calpain as a trigger of severe burn-induced heart injury, which damages the membrane-associated cytoskeleton and membrane integrity. More importantly, our data show that rhTNFR:Fc treatment is an effective strategy to blunt burn-induced heart injury by inhibiting calpain.

TNF-α is an important inflammatory cytokine that is markedly increased after burn injury. The application of its inhibitor to prevent heart dysfunction post burn has not been applied because of the risk of infectious complications [27, 28]. RhTNFR:Fc is a fusion protein consisting of tumor necrosis factor-alpha receptor 2 (TNFR2) bound to the Fc portion of human IgG. In the clinic, rhTNFR:Fc is used to neutralize TNF-α to alleviate the aberrant proinflammatory response during the pathogenesis of rheumatoid arthritis, ankylosing spondylitis, psoriasis, and Crohn’s disease [28–30]. In this study, RhTNFR:Fc injection reduced calpain activity and severe burn-induced heart injury in rats. These data provide evidence for expanding the application of RhTNFR:Fc to protecting the heart after severe burn injury. In addition, our data also reveal calpain as a downstream target of TNF-α but are in contrast to previous studies showing that calpain triggered TNF-α expression via NF-κB activation in sepsis-induced myocardial dysfunction [31, 32]. We suggest that TNF-α interacts with calpain in a reciprocal manner after severe burn injury and that quenching any molecule is an effective strategy for cardioprotection.

Most clinicians and researchers emphasize that hypoperfusion may account for stress cardiomyopathy in burn injury during the early period. Burn injury causes microvascular hyperpermeability, which occurs at burn wounds, as well as in unwounded distant tissues, resulting in massive volume loss, reduced venous return, and hypoperfusion of the heart [33]. Thus, fluid resuscitation regimens have been shown to improve patient recovery during the early post burn period when plasma leakage takes place [34]. On the other hand, cell shock, especially microvascular aberrations, has also received much more attention [33]. In this study, fluid resuscitation was implemented immediately after extensive skin burns, and heart injury was still visible, as manifested by reduced left ventricle function and increased cell death and cTnI release. Furthermore, studies of cultured cardiac cells showed that burn serum directly caused cell injury. These studies strengthen the view that in addition to hypovolemic shock, cell shock also occurs in cardiac cells after burn injury. More importantly, our data suggest that hypoperfusion is not the primary cause of calpain activation or cytoskeletal damage and that calpain/membrane-associated cytoskeletal damage is a new pathway mediating stress cardiomyopathy after severe burn injury.

A typical characteristic of stress cardiomyopathy after severe burn injury is the fragmentation of myocytes [1], which is an indicator of cell membrane rupture, but the pathogenesis of this phenomenon has not been resolved. Accumulating evidence reveals that the structural and functional properties of the plasma membrane depend on membrane-associated skeletal proteins, which are prone to damage by physical and chemical stress [9, 12, 16]. Here, disruption of the membrane-associated cytoskeleton, including spectrin, ankyrin-B, and dystrophin, correlated with heart damage, and the preservation of cytoskeletal proteins and membrane integrity by blocking calpain improved cell survival and heart function. These results suggest that damage to the membrane-associated cytoskeleton or membrane integrity damage caused by calpain is a key factor mediating heart damage after burn injury. Another interesting factor is burn-induced activation of caspase-3. Caspase-3 is a common protein in the apoptosis and pyroptosis pathways. Caspase-3 degrades the DNA repair enzyme PARP-1, causing cell apoptosis [35]. This enzyme also cleaves GSDME and releases the N-terminal domain to perforate the cell membrane, resulting in pyroptosis [36, 37]. In this study, burns damaged the expression of PARP-1 but did not increase the active form of GSDME. These data reveal the occurrence of myocardial apoptosis after burn injury. Furthermore, burn injury increased membrane recruitment of CAPN1, and blocking calpain activity improved the integrity of the plasma membrane and the DNA repair enzyme PARP-1. Thus, it is likely that myocardial apoptosis after burn injury is secondary to calpain-induced membrane integrity damage.

Here, we demonstrated that burn injury activated calpain, as indicated by membrane recruitment of CAPN1 and proteolysis of its substrate α-spectrin. As at least 16 calpain genes have been discovered in eukaryotes, and we identified μ-calpain as a trigger of severe burn-induced heart injury by using siRNA. Calpain has an array of potential roles in heart injury, such as promoting mitochondrial permeability transition pore opening and impairing mitochondrial metabolism during myocardial ischemia/reperfusion stress [38]. In this study, blocking calpain not only preserved the membrane-associated cytoskeleton and membrane integrity but also reduced cell apoptosis, as reflected by decreases in the cytosolic levels of cytochrome c, caspase-3 activities, and PARP-1 degradation. We suggest that μ-calpain cleaves multiple substrates, causing heart injury after severe burn injury.

In addition to stress cardiomyopathy, burn patients also experience acute lung injury and kidney failure [39, 40]. In combination with our previous finding that μ-calpain is involved in acute lung injury after a severe burn [41], we propose a new concept that μ-calpain serves as a common trigger for multiple organ failure and is a potential target for preventing shock in burn victims.

In summary, the present study identifies μ-calpain as a trigger for severe burn-induced heart injury, which involves disruption of the membrane-associated cytoskeleton or membrane integrity. Furthermore, we provide evidence for the application of RhTNFR:Fc to inhibit calpain and protect the heart. Ongoing work will further define the role of μ-calpain in multiple organ failure and potential therapeutic interventions to improve burn patient outcomes.

## Materials and methods

### Chemicals and antibodies

MDL28170 (1146) was purchased from Tocris Bioscience (Bristol, UK). RhTNFR:Fc was provided by Hisun Biopharmaceutical Co. (Hangzhou, China). Antibodies against CAPN1 (2556), cytochrome c (4272), poly ADP-ribose polymerase-1 (PARP-1, 9542), and GAPDH (2118) were obtained from Cell Signaling Technology (Beverly, MA, USA). Antibodies against α-spectrin (BML-FG6090), ankyrin-B (sc-12718), dystrophin (ab15277), and gasdermin E (GSDME, ab215191) were purchased from Enzo Life Sciences (Plymouth Meeting, PA, USA), Santa Cruz Biotechnology (Santa Cruz, CA, USA) and Abcam (Cambridge, MA, USA), respectively. Tetramethylrhodamine-conjugated secondary antibodies (T-2769) were acquired from Thermo Fisher Scientific (Eugene, OR, USA). Assay kits for LDH (KA0878) and caspase-3 activity (K106-100) were provided by Abnova (Taipei, Taiwan) and Biovision (Milpitas, CA, USA). The Mem-PER^TM^ Plus kit (89842) and the Pierce^TM^ BCA protein assay kit (23227) were purchased from Thermo Scientific (Rockford, IL, USA). A small interfering RNA (siRNA) against rat CAPN1 and scramble siRNA were purchased from Santa Cruz Biotechnology (SantaCruz, CA, USA). Evans blue and other chemicals were purchased from Sigma (Shanghai, China).

### Animal and experimental protocol

Male Sprague-Dawley rats weighing 300−350 g were provided by the Laboratory Animal Center of the Fourth Military Medical University (Xi’an, China). All animal procedures conformed to the *Guide for the Care and Use of Laboratory Animals (8th* *edition)* issued by the National Research Council of the United States, and all animal experiments were approved by the Institutional Animal Care and Use Committee of the Fourth Military Medical University. The rats were allocated to experimental groups in accordance with the random number table.

Severe thermal injury, in which the extent of third-degree burns reached approximately 40% of the total body surface area, was induced by routine procedures in our lab [41]. Briefly, the rats were anesthetized by an i.p. injection of 40 mg/kg pentobarbital. The dorsal and lateral back hair in a 8.5 × 5.5 cm area was shaved. The experimental rats were then placed in a wooden mold and immersed in 92 °C water for 18 s to induce skin damage, while the rats in the sham group were immersed in 25 °C water. All rats were quickly dried and resuscitated with an i.p. injection of 50 ml/kg b.w. saline solution. Finally, the rats received an i.p. injection of 0.05 mg/kg b.w. buprenorphine for pain management.

The calpain inhibitor MDL28170 was dissolved in a mixture of dimethyl sulfoxide and saline (1:4) at a concentration of 10 mg/ml, which was used as a stock solution. In MDL28170-treated group, one milliliter of saline supplemented with MDL28170 at a dose of 3 mg/kg b.w. was administered via the tail vein 1 h before burn injury [41], while the burn group was injected with an equal amount of saline and dimethyl sulfoxide (0.2% vol/vol). RhTNFR:Fc was dissolved in saline and injected via the tail vein at a dose of 5 mg/kg b.w. 30 min after the burns were induced. The animal allocations were blinded to the laboratory technician who completed the thermal injury to the rats and the in vivo studies. The rats which died of anesthesia were excluded. Samples were harvested 6 h after burn injury.

### Blood pressure and left ventricular function measurement

At 6 h after burn injury, the rats were anesthetized, a polyethylene catheter (PE-50) was inserted into the right carotid artery, and mean arterial blood pressure (MAP) was measured. Then, the catheter was advanced to the left ventricular cavity, and left ventricular pressure (LVP) was recorded. The peak value of the left ventricular systolic pressure (LVSPmax) and the maximal positive and negative values of the instantaneous first derivative of LVP (±d*p*/d*t*max) were used to reflect heart function, as described previously [42].

### Cell culture, siRNA transfection, and blood plasma treatment

Neonatal cardiac myocytes were isolated from 2- to 3-day-old rat pups [43]. Briefly, the heart tissues were excised, rinsed with saline solution, and digested with multiple rounds of incubation with 1 mg/ml collagenase type II. Non-myocytes were removed by two rounds of plating on culture dishes at 37 °C for 1 h each to enable selective cell attachment. Cardiac myocytes were plated in six-well plates at a density of 3.0 × 10^4^/cm^2^ and maintained in Dulbecco’s modified Eagle’s medium supplemented with 10% fetal bovine serum, 100 IU/ml penicillin, and 100 μg/ml streptomycin in a humid 5% CO_2_ atmosphere at 37 °C.

SiRNA against rat CAPN1 was transfected using TransMessenger Transfection Reagent according to the manufacturer’s protocol, as described previously [44]. The scramble siRNA served as a control. After 12 h, the transfection mixture was replaced with normal culture medium, and the cells were incubated for 24 h. Then, the experimental cells were treated for 4 h with anoxia followed by reoxygenation in KH solution containing (mM) NaCl 125, KCl 5.8, MgSO_4_ 1.2, CaCl_2_ 1.5, NaHCO_3_ 25, and 10% burn plasma, while 10% normal plasma from sham-operated rats served as a control.

### Biochemical assays

At the end of the experiments, blood samples and heart tissues were collected. LDH in the plasma and caspase-3 activity in heart tissue were measured using colorimetric assay kits according to the manufacturer’s instructions [19]. CTnI in the plasma was measured by an established AccuTnI immunoassay (Beckman Coulter, Fullerton, CA), as described previously [19].

### Western blotting

After treatment, left ventricular tissues were excised and washed with saline. Total proteins were prepared with RIPA buffer containing a protease inhibitor cocktail. The cytosolic fraction was extracted with lysis buffer (pH 7.4) containing 250 mM sucrose, 1 mM EDTA, 50 mM Tris-HCl, 1 mM DTT, and protease inhibitors. The plasma membrane fraction was prepared with the Mem-PER^TM^ Plus Kit. Equal amounts of proteins from each sample were separated by SDS–PAGE and transferred to membranes. The membranes were incubated with primary antibodies against cytochrome c, dystrophin, α-spectrin, ankyrin-B, calpain 1, PARP-1, and GSDME (all at 1:1000) overnight at 4 °C, followed by incubation with horseradish peroxidase-conjugated secondary antibodies for 1 h at room temperature. Finally, the membranes were incubated with a chemiluminescent substrate, and the target proteins were visualized with a Quantity One system (Bio–Rad Inc., Hertfordshire, UK), as described previously [17].

### Immunofluorescence analysis

Tissue slices were stained with antibodies against CAPN1 (1:100) and dystrophin (1:100) at 4 °C overnight, followed by incubation with tetramethylrhodamine-conjugated secondary antibodies (1:500). The nuclei were stained with 2 μg/ml DAPI. Finally, fluorescent images were under a confocal laser scanning microscope (Olympus FV1000, Tokyo, Japan) assessed by an examiner who was blinded to the groups. Five fields for each slide were examined in a × 600 field [17].

To examine membrane integrity, 5 ml/kg b. w. 2% Evans blue was injected via the femoral vein. Twelve hours later, the heart was removed and arrested in a modified Tyrode solution containing high potassium chloride (10 mg/ml). Consecutive frozen slices were prepared with a cryostat. Several pairs of 10-μm-thick cross-sectional adjacent slices were randomly selected, washed with acetone, and assessed under a fluorescence microscope equipped with a red 543 nm activation filter and 590 nm barrier filter [17]. The percent area of the stained myocardium was calculated in each field and averaged.

### Statistical analysis

Each independent data with the mean is presented. The sample size was estimated with statistical software PASS on the basis of plasma cTnI data (Kaysville, UT, USA). Statistical analyses were performed using GraphPad 5.0 (GraphPad Software Inc, La Jolla, CA, USA). The data exhibited normal distribution and equality of variance. One-way ANOVA was performed to compare the differences among groups, followed by unpaired Student’s t tests. A two-tailed *P* value less than 0.05 was accepted as statistically significant.

## Supplementary information

Changes of authorship

## Data Availability

The datasets used and/or analyzed during the current study are available from the corresponding author on reasonable request.
