# Supplementary material for: Impairment of μ-calpain activation by rhTNFR:Fc reduces severe burn-induced membrane disruption in the heart
Source: Cell Death Discov. 2022 Jan 10;8:10. doi: 10.1038/s41420-021-00810-8 (PMC8748603; doi:10.1038/s41420-021-00810-8)
Supplement: Supplementary file 1 — Changes of authorship [file 41420_2021_810_MOESM1_ESM.pdf]

# Confirmation for changes of authorship to the manuscript numbered

# CDDISCOVERY-21-2476

This is to certify that all authors have agreed to add Dr. WANG Qiong as a co-corresponding author in the manuscript entitle with “Impairment of μ-calpain activation by rhTNFR:Fc reduces severe burn-induced embrane disruption in the heart”. The following are the e-mail messages for agreements from all authors.

回复：  Yahoo/Inbox

**O(∩_∩)O萝** <1140484703@qq.com>  Sat, Dec 11 at 11:49 AM
**To:** jingjun zhou

Dear Dr. Jing-Jun Zhou

Thank you for emailing to me on this decision. I agree to add Dr. Qiong Wang as a co-author in our manuscript entitled with “Impairment of μ-calpain activation by rhTNFR:Fc reduces severe burn-induced membrane disruption in the heart”.

Best regards

Meng-Shu Cao
2021-12-11

Hide original message

------------------ 原始邮件 ------------------

**发件人:** "jingjun zhou"<jjzhou71@yahoo.com>;
**发送时间:** 2021年12月11日(星期六) 中午11:36
**收件人:** "O(∩_∩)O萝"<1140484703@qq.com>;
**主题:**

Dear Meng-Shu Cao,

Re. MS: CDDISCOVERY-21-2476R1, title: Impairment of μ-calpain activation by rhTNFR:Fc reduces severe burn-induced membrane disruption in the heart

I am writing to inform you that Dr. WANG Qiong is added as a co-corresponding author to this manuscript due to her contributions. Please check through the Author Contributions in the manuscript, and reply this email for a confirmation. If you have any questions, please let me know.

[illegible]

回复：(无主题)  Yahoo/Inbox

**赵霆延** <2110475154@qq.com>  Sat, Dec 11 at 12:26 PM
**To:** jingjun zhou

Dear Dr. Jing-Jun Zhou

Thank you for informing this message to me. I agree to add Dr. Qiong Wang as a co-author in our manuscript, which will be published in Cell Death Discovery.

yours

Ting-Yan Zhao
2021-12-11

---原始邮件---

**发件人:** "jingjun zhou"<jjzhou71@yahoo.com>
**发送时间:** 2021年12月11日(周六) 中午11:37
**收件人:** "2110475154@qq.com"<2110475154@qq.com>;
**主题:** (无主题)

Dear Ting-Yan Zhao,
Re. MS: CDDISCOVERY-21-2476R1, title: Impairment of μ-calpain activation by rhTNFR:Fc reduces severe burn-induced membrane disruption in the heart

I am writing to inform you that Dr. WANG Qiong is added as a co-corresponding author to this manuscript due to her contributions. Please check through the Author Contributions in the manuscript, and reply this email for a confirmation. If you have any questions, please let me know.

Yours

Jing-Jun, ZHOU

(No Subject) 2 Yahoo/Inbox

jingjun zhou Dear Hong-Ting Lu, Re. MS: CDDISCOVERY-21-2476R1, title: Impairn Sat, Dec 11 at 11:33 AM

2016级五年制临床 鲁洪廷 <luhongting19980826@163.com> Sat, Dec 11 at 11:44 AM
To: jingjun zhou

Hi, Jing-Jun Zhou

Nice to hear from you! I believe that Dr. Qiong is deserved as a co-author in our manuscript. I have no questions this decision.

yours

Hong-Ting Lu
2021-12-11

在 2021-12-11 11:33:43，"jingjun zhou" <jjzhou71@yahoo.com> 写道：

Hide original message

> Dear Hong-Ting Lu,
> Re. MS: CDDISCOVERY-21-2476R1, title: Impairment of μ-calpain activation by rhTNFR:Fc reduces severe burn-induced membrane disruption in the heart
>
> I am writing to inform you that Dr. WANG Qiong is added as a co-corresponding author to this manuscript due to her contributions. Please check through the Author Contributions in the manuscript, and reply this email for a confirmation. If you have any questions, please let me know.
>
> Yours
>
> Jing-Jun, ZHOU

Re: (无主题) Yahoo/Inbox

宋志龙 <songzhilong1025@163.com> Sat, Dec 11 at 11:47 AM
To: jingjun zhou

Hi, Prof. Zhou

Thank you for writing to me. Dr. Qiong Wang did do a lot in this project. I agree to add her as a co-author in our manuscript.

yours

Zhi-Long Song
2021-12-11

宋志龙

邮箱：songzhilong1025@163.com

签名由 网易邮箱大师 定制

Hide original message

On 12/11/2021 11:41, jingjun zhou wrote:

鲁涛 <taolu7576@qq.com>
To: jingjun zhou

Sat, Dec 11 at 12:13 PM

Dear Dr. Jing-Jun Zhou

Thank you for emailing to me. I agree to add Dr. Qiong Wang as a co-author in our manuscript.

Merry Christmas

Tao-Lu
2021-12-11

------------------ Original ------------------

**From:** jingjun zhou <jjzhou71@yahoo.com>
**Date:** Sat,Dec 11,2021 11:37 AM
**To:** taolu7576@qq.com <taolu7576@qq.com>
**Subject:** Re:

Dear Tao Lu,
Re. MS: CDDISCOVERY-21-2476R1, title: Impairment of μ-calpain activation by rhTNFR:Fc reduces severe burn-induced membrane disruption in the heart

I am writing to inform you that Dr. WANG Qiong is added as a co-corresponding author to this manuscript due to her contributions. Please check through the Author Contributions in the manuscript, and reply this email for a confirmation. If you have any questions, please let me know.

Yours

Jing-Jun, ZHOU

回复：

Yahoo/Inbox

**18360522@qq.com** <18360522@qq.com>
To: jingjun zhou

Sat, Dec 11 at 12:38 PM

Dear Dr. Jing-Jun Zhou

I have learned of this and I agree to add Dr. Qiong Wang to the list of authors.

Yours

Yun Zheng
2021-12-11

发自我的华为手机

回复：(无主题)

Yahoo/Inbox

**顾晓明** <gxm5683@qq.com>
**To:** jingjun zhou

Sat, Dec 11 at 11:50 AM

Hi, Jing-Jun Zhou

I have no doubt to put Dr. Qiong Wang as a co-author in our manuscript, entitled with “Impairment of μ-calpain activation by rhTNFR:Fc reduces severe burn-induced membrane disruption in the heart”.

Xiao-Ming Gu
2021-12-11

---原始邮件---

**发件人:** "jingjun zhou"<jjzhou71@yahoo.com>
**发送时间:** 2021年12月11日(周六) 中午11:34
**收件人:** "gxm5683@qq.com"<gxm5683@qq.com>;
**主题:** (无主题)

Dear Xiao-Ming Gu,
Re. MS: CDDISCOVERY-21-2476R1, title: Impairment of μ-calpain activation by rhTNFR:Fc reduces severe burn-induced membrane disruption in the heart

I am writing to inform you that Dr. WANG Qiong is added as a co-corresponding author to this manuscript due to her contributions. Please check through the Author Contributions in the manuscript, and reply this email for a confirmation. If you have any questions, please let me know.

Yours

Jing-Jun, ZHOU

Inbox
Unread
Starred
Drafts 1
Sent
Archive
Spam
Trash
Less

Views Hide
Photos
Documents
Subscriptions
Deals
Groceries
Receipts
Travel

Re: Authorship

Yahoo/Inbox

王琼 <xjcardiowangqiong@163.com>
To: jingjun zhou

Sat, Dec 11 at 7:59 PM

Hi Jing-Jun,

I am glad to hear from you. I am very happy to be a co-corresponding author in this manuscript. I hope our manuscript will be published soon.

Yours

Qiong Wang, PhD, MD
2021-12-11
在 2021-12-11 19:46:36，"jingjun zhou" <jjzhou71@yahoo.com> 写道：

Hide original message

Dear Dr. Qiong,

Re. MS: CDDISCOVERY-21-2476R1, title: Impairment of μ-calpain activation by rhTNFR:Fc reduces severe burn-induced membrane disruption in the heart

I am writing to inform you that you have been added as a co-corresponding author to this manuscript due to your contributions. Please check through the Author Contributions in the manuscript, and reply this email for a confirmation. If you have any questions, please let me know.

Yours

Jing-Jun, ZHOU
